# Challenges of neoadjuvant immunotherapy in mismatch repair-deficient/microsatellite-unstable localized colon cancer patients

**DOI:** 10.1016/j.esmoop.2026.106893

**Published:** 2026-03-10

**Authors:** M. Karoui, A. Mariani, J. Taieb

**Affiliations:** 1 Department of Digestive and Oncological Surgery, Paris Cité University, Assistance Publique Hôpitaux de Paris, European Georges Pompidou University Hospital, Paris, France; 2 Department of Digestive Oncology, Assistance Publique Hôpitaux de Paris, European Georges Pompidou University Hospital, Paris, France

**Keywords:** localized colon cancer, non-metastatic colon cancer, MSI/dMMR, neoadjuvant immunotherapy

## Abstract

Despite cumulative evidence on the efficacy and tolerability of neoadjuvant immunotherapy in mismatch repair-deficient (dMMR) colon cancer (CC), it is still hard to draw clear recommendations on its indication in our daily clinical practice. Key clinical trials investigating neoadjuvant or adjuvant immune checkpoint inhibitors (ICIs) in dMMR non-metastatic CC were reviewed. Based on their results, we designed an algorithm in the management of dMMR CC patients. The decision to treat patients with non-metastatic dMMR CC with neoadjuvant or adjuvant ICIs is primarily based on the patient’s clinical status, baseline computed tomography (CT) characteristics of the primary tumor, and the therapeutic objectives.

## Highlights

• ICIs as neoadjuvant treatment in patients with localized dMMR CC is challenging.
• Neoadjuvant ICIs are efficient and well tolerated in CC.
• The decision of neoadjuvant ICIs should be tailored to the patient’s condition, the primary tumor CT scan characteristics and the objectives of treatment.

Microsatellite instability (MSI), caused by a deficiency in the DNA mismatch repair system [mismatch repair-deficient (dMMR)], accounts for ∼10%-15% of locally advanced colon cancers (CCs).^1^ Although early-stage dMMR CC is associated with a favorable prognosis, patients with locally advanced disease, namely T4 and/or N+, remain at significant risk of recurrence, estimated between 15% and 30% at 3 years despite adjuvant chemotherapy.^2^ Neoadjuvant treatment in a perioperative chemotherapy strategy has been shown to improve prognosis of patients with locally advanced CC but failed to provide any oncological benefit for those with a dMMR tumor as compared with upfront colectomy.^3^ Given the demonstrated efficacy of immune checkpoint inhibitors (ICIs) in metastatic dMMR CC,^4^, ^5^, ^6^ neoadjuvant immunotherapy has emerged as a promising approach in locally advanced dMMR CC patients, as the prominent antigen load that exists when the bulk tumor is present at the time of ICI treatment might trigger a broader antitumor immune response, further favored by the presence of intact tumor-draining lymph nodes. In addition, this neoadjuvant ICI-based treatment may improve their prognosis by treating circulating micrometastases and by inducing tumor down-staging, thus improving oncological surgery. It was also suggested that starting with neoadjuvant immunotherapy may provide an opportunity window to assess tumor response and to decide on the next step of action, including a non-operative management (NOM) in selected patients.

Neoadjuvant immunotherapy has been tested in a proof-of-concept study in 2020 in 21 patients with cT3-T4 and/or N+ dMMR CC on initial computed tomography (CT) scan.^7^ Patients were treated with one dose of ipilimumab [anti-cytotoxic T-lymphocyte-associated protein 4 (CTLA-4)] and two doses of nivolumab [anti-programmed cell death protein 1 (PD-1)] 4 weeks before colectomy. At pathological examination, major response (defined as ≤10% of viable residual tumor) was found in 95% of patients and complete response [pathological complete response (pCR)] in 60%.^7^ Several other studies with different ICI regimens including an anti-PD-1 alone or combined with an anti-CTLA-4 or an anti-LAG3 (anti-lymphocyte activation gene-3) target and block the LAG-3 protein, an immune checkpoint receptor on T-cells have confirmed these data with pCR rates ranging from 43% to 93% and showed that combination therapy was associated with higher pCR rates (Table 1).^8^, ^9^, ^10^, ^11^, ^12^, ^13^, ^14^, ^15^, ^16^, ^17^ In a systematic review of 13 studies using a probit model-fitting analysis, a positive association between treatment duration and incidence of complete response (*P* < 0.001; *P* = 0.08 for testing for goodness of fit) has also been demonstrated among patients with non-metastatic CC treated by anti-PD-1 or anti-programmed death-ligand 1 alone.^18^ In addition, the final results of the NICHE study reported on 113 patients showed, after a median follow-up of 26 months, a 3-year disease-free survival (DFS) rate of 100%.^8^ Across these published series, neoadjuvant immunotherapy demonstrated favorable tolerability with timely surgical resection achieved in 95% of patients and a 4%-13.5% risk of grade 3-4 immune-related adverse events (Table 1).

**Table 1:** Key clinical trials investigating neoadjuvant ICIs in MSI-H/non-metastatic colon cancers

Trials	*n*	Treatments	Pathological response rates	Survival outcomes	TRAE rates	Ref
NICHE/NICHE-2 (phase II)	113	Ipilimumab (1 cycle) nivolumab (2 cycles)	MPR: 98% pCR: 69%	3-year DFS 100%	Any grade: 63% Grade 3-4: 4%	^8^
NICHE 3 (phase II)	59	Nivolumab + relatlimab (2 cycles)	MPR: 92% pCR: 68%	1-year DFS 98%	Any grade: 80% Grade 3-4: 10%	^9^
PICC (randomized phase II)	34	Toripalimab ± celecoxib (6 cycles)	MPR: 97% pCR: 76%	1-year DFS 100%	Any grade: 76% Grade 3-4: 6%	^10^
NEST-1 and NEST-2 (phase II)	3	Botensilimab (1 cycle) + balstilimab (1 or 3 cycles)	MPR: 100% pCR: 67%	NA	NA	^11^
NEOPRISM-CRC (phase II)	32	Pembrolizumab (3 cycles)	MPR: 78% pCR: 59%	6-month DFS 100%	NA	^12^
NCT04082571 (phase II)	13	Pembrolizumab (8 cycles)	pCR: 92%	NA	NA	^13^
NCT05890742 (randomized phase Ib)	101	IBI-310 (1 cycle) + sintilimab (2 cycles) versus sintilimab (2 cycles)	pCR: 77% versus 43%	NA	Any grade: 88.5% versus 79.6% Grade 3-4: 6% versus 8% Grade 5: 0% versus 2% (myocarditis)	^14^
IMHOTEP (phase II)	72	Pembrolizumab (1 or 2 cycles)	pCR: 46% to 68%	NA	Any grade: 97.8% Grade 3-4: 13.5% Grade 5: 1% (myasthenia)	^15^
RESET-C (phase II)	85	Pembrolizumab (1 cycle)	MPR: 57% pCR: 44%	NA	Any grade: NA Grade 3: 8% No grade 4 or 5	^16^
UNICORN (phase II)	56	Botensilimab (1 cycle) + balstilimab (2 cycles)	MPR: 100% pCR: 93%	NA	Any grade: 5%	^17^

DFS, disease-free survival; ICIs, immune checkpoint inhibitors; MPR, major pathological response; MSI-H, microsatellite instability-high; NA, not available; pCR, pathological complete response; TRAE, treatment-related adverse event.

However, selecting high-risk stage II and stage III patients who need theoretically more than surgery alone, with baseline CT scan, remains an issue, with 24%-30% of patients over-staged by clinical TNM (radiological) assessment as reported in the PRODIGE22, OPTICAL, FOXTROT and NEOCOL randomized trials, and a real risk of overtreatment in the neoadjuvant setting.^3^^,^^19^, ^20^, ^21^ This CT-based over-staging likely reflects the limited sensitivity of CT imaging for identifying high-risk patients, defined as those with an extramural invasion depth ≥5 mm (T3c-T4) and/or nodal involvement (N+).^22^ Although differences in CT imaging features between mismatch repair-proficient and dMMR CCs have been reported, particularly regarding primary tumor and lymph node size and heterogeneity,^23^ substantial inter-institutional variability in image interpretation underscores the need for standardized imaging protocols and reporting criteria in this setting.^24^ Magnetic resonance imaging (MRI), CT colonography, and positron emission tomography-CT are currently being evaluated to improve staging accuracy, with promising results.^25^, ^26^, ^27^ Although these approaches are still in an exploratory phase, artificial intelligence-based imaging techniques, including radiomics and machine-learning models, are expected to become valuable tools in future clinical workflows, enabling the identification of high-risk patients who may benefit from neoadjuvant treatment.

Beyond patients with locally advanced CC (i.e. unequivocal cT4 or unresectable), who often require more extensive surgery and/or carry a higher risk of incomplete tumor resection, and in whom neoadjuvant ICIs may represent a valid option, the potential benefit of neoadjuvant ICIs in other subsets of dMMR CC may be outbalanced by their favorable prognosis, together with the non-negligible risk of over-staging on CT and unnecessary long-lasting immune-related adverse events. For those undergoing upfront surgery and found stage III at pathological examination, the addition of adjuvant atezolizumab for 12 months to standard FOLFOX chemotherapy for 6 months has been recently reported to improve 3-year DFS as compared with FOLFOX alone in the phase III ATOMIC trial (86% and 76%, respectively; HR 05, CI 0.34-0.72, *P* < 0.0001).^28^ However, it should be emphasized that patients with high-risk stage II CC (pT4N0) were not eligible for the ATOMIC trial; therefore, adjuvant chemotherapy combined with ICIs cannot be recommended for this subgroup of patients.

**Figure 1:**
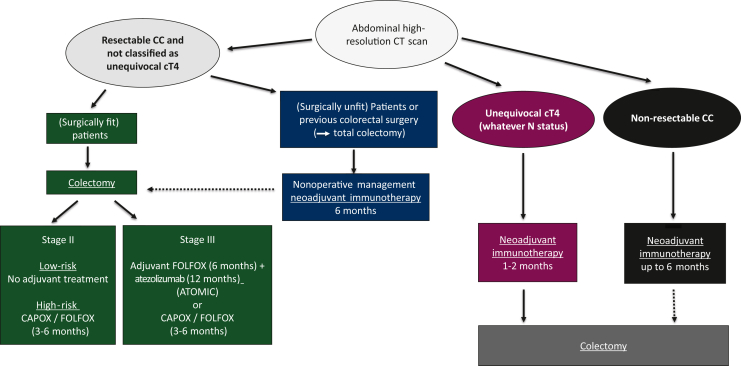
**Algorithm proposal of management of dMMR non-metastatic colon cancer.** CC, colon cancer; CT, computed tomography; dMMR, mismatch repair-deficient.

Despite cumulative evidence on the efficacy and tolerability of neoadjuvant ICIs in dMMR/MSI CC, it is still hard to draw clear recommendations on its indication in our daily clinical practice. In our view, the decision-making process depends mainly on the patient’s condition, the characteristics of the baseline CT scan and the treatment objectives (Figure 1):

• In surgically fit patients with resectable primary CC not classified as unequivocal cT4 or unresectable on baseline CT scan, upfront surgery remains the standard of care and although adjuvant CAPOX/FOLFOX 3-6 months remains the standard of care in current guidelines^29^ for high-risk stage II and stage III disease, FOLFOX (6 months) + atezolizumab (12 months) should be discussed for stage III patients following the ATOMIC study results.
• In patients with primary CC judged as unequivocal cT4 (whatever N status) or unresectable on initial CT scan, the aim of neoadjuvant immunotherapy is to undergo an R0 resection if a sufficient downsizing occurs. To avoid prolonging the time to surgery and to reduce the risks of treatment-related adverse events (obstruction, perforation, bleeding, immune related), a limited duration of neoadjuvant immunotherapy favoring a combination therapy may represent a valid option. Tumor response should be assessed by a CT scan and carcinoembryonic antigen dosage, 6-8 weeks after treatment start and surgery planned as soon as possible.
• If the objective is organ preservation and NOM, clinical complete response becomes the goal of neoadjuvant immunotherapy, and a longer duration of treatment favoring monotherapy, with an increased time to response assessment, may represent the preferred option as reported in dMMR rectal cancer.^30^ Although NOM is well established in the treatment of rectal cancer, there are notable differences with CC. Firstly, colectomy is a less demanding surgical procedure with lower morbidity, carries a lower risk of definitive stoma and generally does not negatively impact long-term functional, genitourinary functions and quality of life than rectal surgery. Secondly, there are currently no validated criteria for defining clinical complete response (cCR) in CC. Thirdly, although the follow-up for rectal cancer is standardized including pelvic MRI, digital examination and rectoscopy, there are no established surveillance protocols to support a NOM approach in CC and repeated complete colonoscopy with general anesthesia may represent a major limitation. Taken together with the fact that most of the dMMR CC patients can be cured by surgery alone, the NOM of dMMR CC cannot be considered evidence-based at this time and should be discussed with caution and only proposed in a Multidisplinary Team meeting to selected frail or elderly patients with severe comorbidities who are surgically unfit. NOM should also be discussed in patients with previous colectomy (Lynch syndrome) for whom a sub-complete colectomy may result in a significant quality-of-life deterioration. In cases where neoadjuvant ICI is administered with the intention of pursuing a NOM strategy, failure to achieve a cCR should prompt a reassessment of the patient’s surgical fitness to re-consider a curative-intent surgery. The ongoing French PREMICES trial (NCT06646445) will answer these questions by randomizing surgery and NOM in dMMR CC after neoadjuvant immunotherapy and will bring useful information on monitoring modalities (imaging, endoscopic and pathological findings) and their results. New surveillance modalities such as circulating tumor DNA may also be of interest in this context to enable optimal non-invasive surveillance of NOM patients in the future.

Waiting for the results of large randomized ongoing phase III studies, such as AZUR-02 (NCT05855200), the integration of neoadjuvant ICIs into the treatment algorithm of non-metastatic dMMR CC patients represents a paradigm shift that requires currently a nuanced and objective-based approach. Critical questions remain unanswered regarding optimal treatment duration, choice between single-agent or doublet immunotherapeutic regimens, appropriate patient selection (including improved imaging and biomarkers), the added value of post-operative ICIs, long-term oncological outcomes, surveillance programs and immune-related toxicity, together with the financial toxicity of these expensive treatments. As such, there is a pressing need for well-designed prospective clinical trials specifically addressing these open questions.
